# Effects of curcuminoids identified in rhizomes of *Curcuma longa* on BACE-1 inhibitory and behavioral activity and lifespan of Alzheimer’s disease *Drosophila* models

**DOI:** 10.1186/1472-6882-14-88

**Published:** 2014-03-05

**Authors:** Xue Wang, Jun-Ran Kim, Seong-Baek Lee, Young-Joon Kim, Moon Young Jung, Hyung-Wook Kwon, Young-Joon Ahn

**Affiliations:** 1 Entomology Major, Department of Agriculture Biotechnology, Seoul National University, Seoul 151-921, Republic of Korea; 2 WCU Biomodulation Major, Department of Agricultural Biotechnology, Seoul National University, Seoul 151-921, Republic of Korea; 3 Cellumed Co. Ltd, Geumocheon-gu, Seoul 153-782, Republic of Korea; 4 School of Life Sciences, Gwangju Insititute of Science and Technology, Gwangju 500-712, Republic of Korea

**Keywords:** Alzheimer’s disease, *Drosophila melanogaster*, *Curcuma longa*, Curcuminoids, BACE-1, Structure–activity relationship

## Abstract

**Background:**

Alzheimer’s disease (AD) is the most common type of presenile and senile dementia. The human β-amyloid precursor cleavage enzyme (BACE-1) is a key enzyme responsible for amyloid plaque production, which implicates the progress and symptoms of AD. Here we assessed the anti-BACE-1 and behavioral activities of curcuminoids from rhizomes of *Curcuma longa* (Zingiberaceae), diarylalkyls curcumin (CCN), demethoxycurcumin (DMCCN), and bisdemethoxycurcumin (BDMCCN) against AD *Drosophila melanogaster* models.

**Methods:**

Neuro-protective ability of the curcuminoids was assessed using *Drosophila melanogaster* model system overexpressing BACE-1 and its substrate APP in compound eyes and entire neurons. Feeding and climbing activity, lifespan, and morphostructural changes in fly eyes also were evaluated.

**Results:**

BDMCCN has the strongest inhibitory activity toward BACE-1 with 17 μM IC_50_, which was 20 and 13 times lower than those of CCN and DMCCN respectively. Overexpression of APP/BACE-1 resulted in the progressive and measurable defects in morphology of eyes and locomotion. Remarkably, supplementing diet with either 1 mM BDMCCN or 1 mM CCN rescued APP/BACE1-expressing flies and kept them from developing both morphological and behavioral defects. Our results suggest that structural characteristics, such as degrees of saturation, types of carbon skeleton and functional group, and hydrophobicity appear to play a role in determining inhibitory potency of curcuminoids on BACE-1.

**Conclusion:**

Further studies will warrant possible applications of curcuminoids as therapeutic BACE-1 blockers.

## Background

Alzheimer’s disease (AD) is the most common cause of presenile and senile dementia in developed and developing countries [1,2]. The worldwide prevalence of AD was 26.6 million in 2006, and this figure is projected to grow up to 106.8 million by 2050 [3]. AD is a devastating neurodegenerative disorder of the brain characterized by accumulation and deposition of amyloid β (Aβ) peptide, which are generated by sequential proteolytic processing of transmembrane amyloid precursor protein (APP) by two enzymes, β-secretase (β-site APP cleaving enzyme or BACE-1) and γ-secretase, in the amyloidogenic processing pathways [4,5]. Besides acetylcholinesterase (AChE), BACE-1 is also considered as a key therapeutic target for prevention and treatment of AD [6,7]. Transgenic models of AD in *Drosophila melanogaster* by driving Aβ production in the central nervous system and retina of the fly have been developed to gain insight into mechanism of AD and to illuminate potential therapeutic approaches [8-10]. Although many peptides and heterocyclic compounds have been designed and evaluated as BACE-1 inhibitors [11-13], none of them have been successfully developed as AD treatment drugs. There is, therefore, a critical need for the development of new improved anti-AD agents.

Plant secondary substances have been suggested as potential alternatives for AD therapy largely because plants constitute a potential source of bioactive chemicals that have been perceived by the general public as relatively safe and often act at multiple and novel target sites [14,15]. These potential new anti-AD products can be applied to humans in the same manner as conventional anti-AD drugs. Much effort has been focused on them as potential sources of commercial anti-AD products for prevention or treatment of AD. BACE-1 or AChE inhibitors from plants have been well reviewed [13,16] respectively. Recently, plants in the family Zingiberaceae have drawn attention because they contain anti-AD principles [17,18]. The rhizomes of turmeric, *Curcuma longa* L., are not only important as a spice or flavoring, but they have also been prescribed for indigestion, hepatitis, jaundice, diabetes, atherosclerosis and bacterial infection [19-21]. Curcumin, an active ingredient of *C. longa*, has been proposed to alleviate Aβ toxicity in transgenic human Aβ and human tau flies by reducing the pre-fibrillar/oligomeric species of Aβ [18].

In the current study, an assessment was made of the BACE-1 inhibitory activity of the three curcuminoid compounds isolated from *C. longa* rhizome (curcumin, demethoxycurcumin, bisdemethoxycurcumin), commercially available curcuminoid compound (tetrahydrocurcumin), a natural BACE-1 inhibitor (EGCG) [22], and a cell-permeable isophthalamide compound (BACE-1 Inhibitor IV) [23], using a fluorescence resonance energy transfer (FRET) enzyme assay. The effects of the two curcuminoids (curcumin and bisdemethoxycurcumin) on feeding, climbing, and life span as well as morphological changes in the compound eyes of *D. melanogaster* which express human APP and BACE-1 genes within the developing nervous system and compound eyes were compared with those of Inhibitor IV. The mode of anti-AD action and quantitative structure–activity relationship (QSAR) of the curcuminoids also are discussed.

## Methods

### Experimental groups

**Figure 1:**
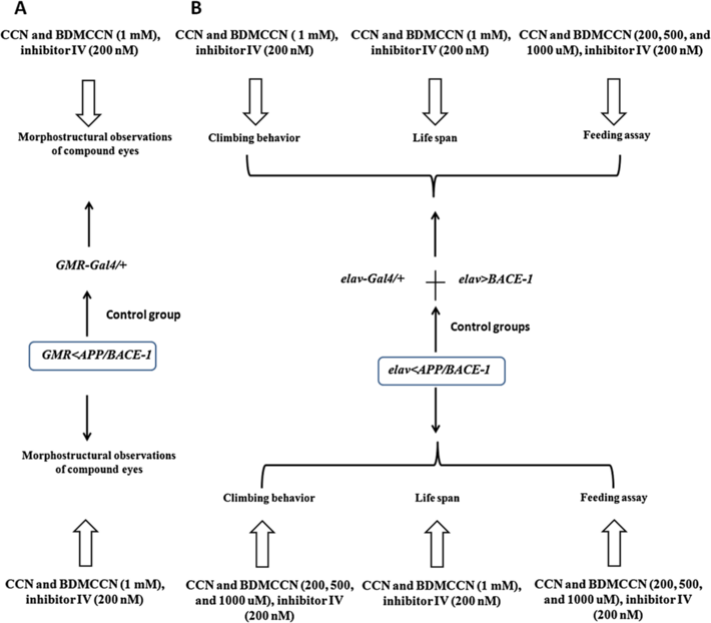
**The schematism of experimental fly groups included in our study.***GMR-Gal4* drives target human APP and BACE-1 genes expression in flies’ compound eyes, and morphostructural abservation changes of these compound eyes with the supplementation of compounds with different concentration, *GMR-Gal4/+* as the control group **(A)**, *elav-Gal4* drives target genes co-expression in flies’ nervous system, observation behaviors including climbing, life span and feeding assay with the supplementation of compounds with different concentration, *elav-Gla4/+* and *elav < BACE-1* used as control groups **(B)**.

In our experiment, three curcuminoids isolated from *Curcuma longa* and one commercial curcuminoid were tested *in vitro*. Among these compounds, only two curcuminoids, curcumin and bisdemethoxycurcumin, were tested to flies, because the BACE-1 inhibitory activity of demethoxycurcumin *in vitro* lied between curcumin and bisdemethoxycurcumin. With this issue, demethoxycurcumin was removed from the *in vivo* study. The experimental groups tested for *in vivo* study were illustrated in Figure 1. *GMR-Gal4* drives target human APP and BACE-1 genes expression in flies’ compound eyes, and morphostructural changes of these compound eyes with the supplementation of compounds with different concentration were observed, *GMR-Gal4/+* was used as the control group (Figure 1A), *elav-Gal4* drives target genes co-expression in flies’ nervous system, behaviors including climbing, life span and feeding assay with the supplementation of compounds with different concentration were tested, *elav-Gla4/+* and *elav < BACE-1* were used as control groups (Figure 1B).

### Instrumental analysis

^1^H and ^13^C NMR spectra were recorded in CD_3_CN on a Bruker AVANCE 600 spectrometer (Karlsruhe, Germany) using tetramethylsilane as an internal standard, and chemical shifts were given in δ (ppm). UV spectra were obtained in MeCN on a Kontron UVICON 933/934 spectrophotometer (Milan, Italy) and mass spectra on a Jeol JMS-DX 303 spectrometer (Tokyo, Japan). Merck silica gel (0.063–0.2 mm) (Darmstadt, Germany) was used for column chromatography. Merck pre-coated silica gel plates (Kieselgel 60 F_254_) were used for analytical thin-layer chromatography (TLC). Merck preparative thin-layer chromatography plates (2 mm thickness) and Biotage Isolera one medium-pressure liquid chromatography (MPLC) (Uppsala, Sweden) were used for isolation of active principles.

### Materials

The four commercially-available pure organic curcuminoids examined in this study are listed in Table 1, along with their sources. For the QSAR analysis, values of molecular weight (MW), hydrophobic parameter (log *P*) and steric effects for the test curcuminoids were obtained from ChemDraw Ultra 10.0 (Cambridge Soft Corporation, Cambridge, MA) and recorded in Table 1. Molecular refraction (MR) was used as the parameter for describing steric effects. Structures of these curcuminoids are given in Figure 1. EGCG was purchased from Sigma-Aldrich (St. Louis, MO), Inhibitor IV, and Acid red were purchased from Merck (Darmstadt, Germany), and Amresco (Cochran Road Solon, OH), respectively. Recombinant human BACE-1 and fluorogenic peptide substrate Mca-SEVNLDAEFRK (Dnp) RR-NH_2_ were purchased from R&D system (Minneapolis, MN). All of the other chemicals and reagents used in this study were of analytical grade quality and available commercially.

**Table 1:** Values of physical parameters of four curcuminoids examined in this study

**Compound**	**MW**^**a**^	**log** ***P***^**b**^	**MR**^**c**^	**Source**^**d**^
Curcumin	368	2.92	104	S-A
Demethoxycurcumin	338	3.08	98.78	S-A
Bisdemethoxycurcumin	308	3.32	92.10	S-A
Tetrahydrocurcumin	372	2.73	100.63	S-A

^a^Molecular weight.

^b^Hydrophobic parameter expressed as the log of the octanol/water partition coefficient.

^c^Parameter for steric effects as described using molecular refraction.

^d^Purchased from Sigma-Aldrich (St. Louis, MO).

### Plants

The rhizomes of *C. longa* were purchased from Boeun medicinal herb shop, Kyoungdong market (Seoul, Republic of Korea (ROK)). A voucher specimen (CL-R1) was deposited in the Research Institute for Agriculture and Life Science, College of Agriculture and Life Sciences, Seoul National University.

### Fly stocks

Flies were cultured in a standard cornmeal agar medium at 25°C and 70% relative humidity (RH) under a 12:12 h light:dark cycle. Following fly stocks were obtained from Bloomington Stock Center at Indiana University: *w1118* (stock number, 3605), *UAS-BACE-1, UAS-APP* (33797), *UAS-BACE-1* (29877), *elav-GAL4* (8760), and *GMR-GAL4* (1104). The GAL4/UAS system was employed for the overexpression of desired genes in a specific tissue of the fly. The transgenic fly stock *UAS-BACE-1* (29877) in our study was also used in previous study [10]. The characterizations of trans-human gene APP and BACE-1 flies as a reliable AD model were presented in results section.

### Extraction and isolation

The dried rhizomes of *C. longa* (1.2 kg) was pulverized and extracted with methanol (3 × 5 L) at room temperature for 2 days and filtered. The combined filtrate was concentrated under vacuum at 40°C to yield ~105.4 g of a dark yellowish red tar. The extract (100 g) was sequentially partitioned into hexane- (31 g), chloroform- (6.55 g), ethyl acetate- (49 g), butanol- (3.95 g), and water-soluble (9.5 g) portions for subsequent bioassay. The organic solvent-soluble portions were concentrated to dryness by rotary evaporation at 40°C and the water-soluble portion was freeze-dried. For isolation of active principles, 2 mg/ml of each *C. longa* rhizome-derived material was tested in a FRET enzyme assay as described previously [24].

The chloroform-soluble fraction (5 g) was most active and MPLC was performed using a Biotage Isolera apparatus equipped with a UV detector at 254 nm and a column cartridge SNAP (100 g silica gel) with column volume 132 ml. Separation was achieved with a gradient of chloroform and methanol [(100:0 (500 ml), 96:4 (1800 ml), 90:10 (400 ml), 80:20 (400 ml) and 0:100 (500 ml) by volume] at a flow rate 50 ml/min to provide 17 fractions (each about 180 ml). Column fractions were monitored by TLC on silica gel plates developed with chloroform and methanol (95:5 by volume) mobile phase. Fractions with similar *R*_f_ values on the TLC plates were pooled. Spots were detected by spraying with 2% H_2_SO_4_ and then heating on a hot plate. Fractions 8 to 11 (1.5 g) were purified by preparative TLC with chloroform and methanol (95:5 by volume) to yield two active principles **1** (41.7 mg, *R*_f_ = 0.47) and **2** (27.2 mg, *R*_f_ = 0.42).

The active ethyl acetate-soluble fraction (500 mg) was chromatographed on a 70 × 1.5 cm silica gel (70 g) column by elution with a gradient of chloroform and methanol [(100:0 (250 ml), 99:1 (550 ml), 98:2 (300 ml), 97:3 (300 ml), 96:4 (200 ml), 90:10 (100 ml) and 0:100 (800 ml) by volume] to afford eight fractions (each about 300 ml). The active fractions 5 to 6 (34.3 mg) were pooled and recrystallized in acetone at −20°C to afford an active principle **3** (13.2 mg).

### FRET enzyme assay

The previous method [24] was used with a slight modification to assess the BACE-1 inhibitory activity of all compounds. In brief, the assay mixtures containing 1 μl of 0.5 μg/μl recombinant human BACE-1, 0.75 μl of 2.5 μg/μl fluorogenic peptide substrate, 47.25 μl of 50 mM sodium acetate (pH 4.5), and the constituents (0.1–2000 μg/ml) in 2% dimethyl sulfoxide (DMSO) were preincubated at 25°C for 1 h followed by adding 16.6 μl of 2.5 M sodium acetate to stop the reaction. Natural BACE-1 inhibitors (EGCG) and Inhibitor IV served as standard references and were similarly prepared. The fluorescence intensity was measured using a Molecular Devices SpectraMAX Gemini XS plate reader (Sunnyvale, CA) at 355 nm excitation and 405 nm emission at room temperature. The inhibition percentage was calculated with the following equation: % inhibition = 100 – [(*Fs* – *Fb*)/(*Fn* – *Fb*)] × 100, where *Fs* was the fluorescence of sample, *Fb* was the fluorescence of the mixture of substrate and DMSO without BACE-1 enzyme, and *Fn* was the fluorescence of the mixture of BACE-1 enzyme, substrate, and DMSO.

### Histological analysis

Cason’s trichome staining was performed as described before [25], In brief, the heads of 5-day-old male flies were fixed in 4% paraformaldehyde buffer solution (pH, 7.4) at 4°C overnight, after which, paraffin-embedded preparations of the fly heads were sectioned at 10 μm thickness by using a HM 340E rotary microtome (Thermo Scientific Microm, Walldorf, Germany). Sections were dried at 40°C overnight and subsequently dewaxed with CitriSolv (Fisher Scientific, Fair Lawn, NJ) and rehydrated with a series of ethanol to phosphate-buffered saline solution. Rehydrated paraffin sections were soaked into Cason’s trichrome stain for 15 min, and slides were gently swashed in tap water with subsequent wash in distilled water three times. Excess of water was removed and samples were mounted with mounted with a Vectorshield H-1000 mounting medium (Vector Laboratories, Burlingame, CA).

For Congo red staining, sections were dewaxed and then stained in Congo red solution for 12 min, after which sections were rinsed in tap water and dehydrated in 50, 70% ethanol for 1 min, followed by the incubation in 100% ethanol for 4 min. Slides were dried and mounted with mounting medium. Images were observed and captured using EZ4 HD equipped with an Integrated 3.0 Mega-Pixel CMOS camera (Leica, Heerbrugg, Switzerland).

### RT-PCR analysis of human APP and BACE-1 genes in transgenic fly

Semi-quantitative RT-PCR was performed to assess the expression levels of human APP and BACE-1 genes in transgenic fly. Total RNA was extracted from the 30–35 heads of 10-day-old male flies using Trizol (Invitrogen Corparation, Carlsbad, CA). RNA and primers were subjected to RT-PCR by using AccuPower RT-PCR Premix (Cat. No. K-2055) (Bioneer, Alameda, CA). This premix contained optimal concentration of all the components necessary for cDNA synthesis and RTase inactivation, as well as amplification in a single 0.2 ml tube. PCR amplifications were performed with specific primers in a total volume of 20 μl containing 2 μl of forward and reverse primer mixture (10 pmol of each primer), 1 μg RNA and DEPC water. The mixture was used for the amplification after initial denaturation at 95°C and 32 cycles (95°C for 30 s, 60°C for 30 s, and 72°C for 30 s). PCR products were visualized by 2% agarose gel electrophoresis containing ethidium bromide. The primer sequences were as follows: for human APP, 5′-GCCGTGGCATTCTTTTGGGGC-3′(forward) and 5′-GTGGTCAGTCCTCGGTCGGC-3′ (reverse) [26]; for human BACE-1, 5′-GCAGGGCTACTACGTGGAGA-3′ (forward) and 5′-GTATCCACCAGGATGTTGAGC-3′ (reverse) [27]. RP49, which encodes the *Drosophila* ribosomal protein 49, was used as an internal standard and reference gene using forward and reverse primer pairs 5′-CTGCTCATGCAGAACCGCGT-3′ and 5′-GGACCGACAGCTGCTTGGCG-3′ [26], respectively. The ethidium bromide stained gel image was digitalized using the Molecular Imager Gel Doc XR System (Bio-Rad, Hercules, CA), and calculated by densitometry [28]. Results are presented as relative mRNA expression of each gene to that of RP49 mRNA.

### Light microscopy and scanning electron microscopy of adult eyes

Flies were cultured from egg stage on 94 × 25 mm polystyrene vials containing standard media supplemented with each test compound as stated previously. Whole adult flies (1, 24, and 36 days old) were anesthetized in ice and were put on a microscope slide at room temperature for light microscopy. Morphostructural observations of eye were made with a Leica EZ4HD equipped with an Integrated 3.0 Mega-Pixel CMOS camera with 35 × magnification (Hicksville, New York).

For scanning electron microscopy (SEM), ice-anesthetized flies (36 days old) were attached to a copper mount using silver paint as a conducting adhesive. They were then put directly into the viewing chamber of a scanning electron microscope without prior coating [29]. The external surface eye morphology was visualized by a Carl Zeiss Supra 55VP field-emission scanning electron microscope (Oberkochen, German) at 15 kV.

### Life span assay

Groups of 200 newly eclosed male flies equally distributed in 10 vials were incubated in media supplemented with 1 mM CCN, 1 mM BDMCCN, or 200 nM inhibitor IV in 0.1% DMSO. Controls received 0.1% DMSO only. Survivors were transferred to fresh media vials every 4 days. The median survival time (T_1/2_) as the time when the survivor function equals 50% was determined because median survivorship reflects a more reliable metric than the mean survival time [10]. All treatments were replicated 10 times.

### Climbing assay

For the climbing activity, we followed procedure described previously [9,30]. Flies were collected at eclosion and cultured in groups of 20 flies in media supplemented with 1 mM CCN, 1 mM BDMCCN, or 200 nM inhibitor IV in 0.1% DMSO. Flies over-expression APP/BACE-1 were treated with different concentrations (200, 500, and 1000 μM) of curcuminoids, based on our preliminary test results and previous studies [17,31]. Control flies received media with 0.1% DMSO. Twenty flies were placed in an empty polystyrene *D. melanogaster* vial (95 mm tall × 24 mm diameter) conjoined with other vial on top and manually banged twice. After 20 s, we counted flies that climbed and crossed the 9.5 cm line from the bottom and calculated the climbing index as the percentage of those relative to the total number of test flies. All trials were replicated five times.

### Feeding assay

The adult feeding assay was performed according to previous study [32] with minor modifications. Flies were collected at eclosion and aged in groups of 15 males and 15 females with culture media for 3 days, and then starved for 20 h in vials containing 3 layers of a Whatman no. 2 filter paper (Maidstone, UK) soaked with distilled water. After starvation, flies were transferred onto vials containing the media (with 0.2% Acid red) supplemented with CCN (200, 500, or 1000 μM), BDMCCN (200, 500, or 1000 μM), or inhibitor IV (200 nM) in 0.1% DMSO. Controls were fed with media containing 0.2% Acid red and 0.1% DMSO. After 2 h of feeding, flies were anesthetized, and their abdomens were isolated and homogenized in 1 ml of distilled water. After centrifugation (5000 rpm, 25°C, 5 min), the optical density (OD) of the supernatant was measured at 505 nm. The OD values were as the index of the amount of food taken by flies [33]. All treatments were replicated three times.

### Data analysis

Concentrations of the test compounds causing 50% loss of BACE-1 (IC_50_) were calculated using GraphPad Prism 5.1 software (San Diego, CA). The IC_50_ values for each *D. melanogaster* line and their treatments were considered to be significantly different from one another when their 95% confidence limits (CL) failed to overlap. All data are presented as mean ± standard error, and the significance between means was determined using one-way or two-way analysis of variance (ANOVA) statistical test (GraphPad Prism 5.1 software; San Diego, CA). Statistical analysis for survival data were carried out using the Bonferroni post tests (GraphPad Prism 5.1 software).

## Results

### FRET bioassay-guided fractionation and isolation of curcuminoids

Fractions obtained from the solvent hydrolyzable of the methanol extract of *C. longa* rhizomes were examined for inhibitory activity against human BACE-1 using a FRET-based enzyme assay (Table 2). At a concentration of 2 mg/ml, both the methanol extract and chloroform-soluble fractions suppressed completely activity of BACE-1. At 1 mg/ml, the chloroform-soluble fraction was the most potent inhibitory material, followed by the ethyl acetate-soluble fraction. Low and no inhibition were produced by the butanol- and water-soluble fractions, respectively. Therefore, the chloroform- and ethyl acetate-soluble fractions were subjected to further purification steps to identify inhibitory constituents for BACE-1.

**Table 2:** **BACE-1 inhibitory in vitro activity of fractions obtained methanol extract of *Curcuma longa *** **rhizomes**

**Material**	**% inhibition**		
	**2 mg/ml**	**1 mg/ml**	**0.5 mg/ml**
Methanol extract	100	65	57
Hexane-soluble fraction	85	48	43
Chloroform-soluble fraction	100	81	71
Ethyl acetate-soluble fraction	84	76	70
Butanol-soluble fraction	82	26	3
Water-soluble fraction	0	0	0

**Figure 2:**
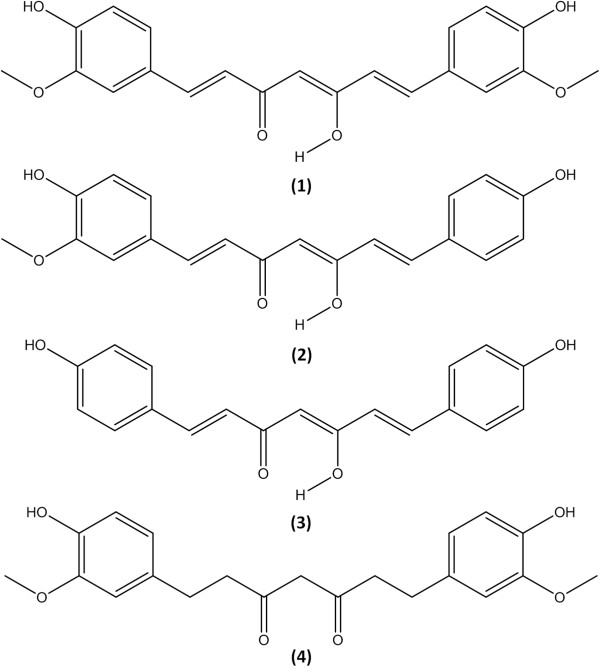
**Structures of the curcuminoids.** Curcumin (**1**), demethoxycurcumin (**2**), bisdemethoxycurcumin (**3**), and tetrahydrocurcumin (**4**).

FRET assay-guided fractionation of *C. longa* rhizome extract afforded three active principles identified by spectroscopic analyses, including MS and NMR. The three active principles were curcumin **(1)**, demethoxycurcumin **(2)**, and bisdemethoxycurcumin **(3)** (Figure 2). Curcumin (**1**) was identified on the basis of the following evidence: brightly yellow colored powder. UV (MeCN): λ _max_ nm 430. EI-MS (70 eV), *m/z* (relative intensity): 368 [M^+^] (100), 350 (67), 272 (23), 231 (24), 217 (25), 191 (48), 190 (60), 177 (93), 145 (26), 137 (44) (Additional file 1: Figure S1). ^1^H NMR (CD_3_CN, 600 MHz): δ 3.31 (6H, s), 5.91 (1H, s), 6.70 (2H, d, *J* = 15.78), 6.86 (2H, d, *J* = 8.10), 7.14 (2H, d, *J* = 8.19), 7.26 (2H, d, *J* = 1.56), 7.58 (2H, d, *J* = 15.78), 9.79 (2H, s), 16.41 (1H, bs) (Additional file 2: Figure S2). ^13^C NMR (CD_3_CN, 600 MHz): δ 56. 8 q, 56.8 q, 102.2 d, 112.1 d, 112.1 d, 116.8 d, 116.8 d, 122. 9 d, 122.9 d, 124.4 s, 124.4 s, 128.7 d, 128.7 d, 142.1 d, 142.1 d, 149.3 s, 149.3 s, 150.5 s, 150.5 s, 206.7 s, 206.7 s (Additional file 3: Figure S3). Demethoxycurcumin **(2)**: yellow-orange amorphous powder. UV (MeCN): λ_max_ nm 430. EI-MS (70 eV), *m/z* (relative intensity): 338 [M^+^] (100), 320 (83), 191 (60), 190 (55), 177 (63), 150 (32), 147 (98), 140 (46), 57 (33) (Additional file 4: Figure S4). ^1^H NMR (CD_3_CN, 600 MHz): δ 3.31 (3H, s), 5.92 (2H, s), 6.64 (1H, d, *J* = 15.84), 6.67 (2H, d, *J* = 15.84), 6.85 (1H, d, *J* = 8.40), 6.87 (2H, d, *J* = 8.64), 7.14 (2H, d, *J* = 6.48), 7.26 (1H, d, *J* = 1.74), 7.53 (2H, d, *J* = 8.64), 7.58 (2H, d, *J* = 15.84) (Additional file 5: Figure S5). ^13^C NMR (CD_3_CN, 600 MHz): δ 56.8 q, 101.8 t, 111.6 d, 116.7 d, 117.3 d, 122.2 d, 122. 4 d, 124.5 d, 128.3 s, 130.6 d, 131.1 d, 131.1 d, 135.2 s, 141.2 d, 141.5 d, 147.1 s, 148.9 s, 158.4 s, 206.3 s, 206.3 s (Additional file 6: Figure S6). Bisdemethyoxycurcumin **(3)**: yellow crystal. UV (MeCN) λ_max_ nm 412. EI-MS (70 eV), *m/z* (relative intensity): 308 [M^+^] (52), 290 (33), 202 (20), 161 (30), 160 (44), 147 (100), 120 (23), 119 (25), 107 (31) (Additional file 7: Figure S7). ^1^H NMR (CD_3_CN, 600 MHz): 5.93 (1H, s), 6.81 (4H, d, *J* = 8.64), 7.48 (4H, d, *J* = 8.58), 7.56 (4H, d, *J* = 15.78), 8.90 (2H, s), 16.40 (1H, bs) (Additional file 8: Figure S8). ^13^C NMR (CD_3_CN, 600 MHz): δ 102.2 t, 117. 3 d, 117.3 d, 117.3 d, 117.3 d,122.6, d, 122.6 d, 128.2 d, 128.2 d, 131.5 d, 131.5 d, 131.5 d, 131.5 d, 142.0 d, 142.0 d, 161.0 s, 161.0 s, 185.1 s, 185.1 s (Additional file 9: Figure S9). The interpretations of proton and carbon signals of compounds **1**, **2**, and **3** were largely consistent with previously described [34].

### *In vitro* BACE-1 inhibitory activity of curcuminoids

The BACE-1 inhibitory activity of all compounds was likewise compared using *in vitro* FRET-enzyme assay (Table 3). Based on IC_50_ values, BDMCCN was 20 and 13 times more potent at inhibiting BACE-1 than CCN and DMCCN. The inhibitory activity of DMCCN was significantly different from that of CCN. THCCN was ineffective. Overall, these curcuminoids were significantly less potent at inhibiting BACE-1 than inhibitor IV. BDMCCN was significantly more active than EGCG.

**Table 3:** Human BACE-1 inhibitory in vitro activity of four curcuminoids, two phytochemicals and BACE-1 inhibitor IV

**Compound**	**IC**_**50**_**, μM (95% CL)**	**Slope ± SE**
Curcumin	340 (296–391)	0.7 ± 0.03
Demethoxycurcumin	217 (197–240)	1.0 ± 0.05
Bisdemethoxycurcumin	17 (14–20)	1.9 ± 0.21
Tetrahydrocurcumin	> 2000	
(−)-Epigallocatechin gallate	82.01(72.59–92.66)	0.9 ± 0.04
BACE-1 inhibitor IV	0.085 (0.075–0.095)	1.2 ± 0.07

### Characterization of trans-human APP and BACE-1 genes fly as a reliable AD model

**Figure 3:**
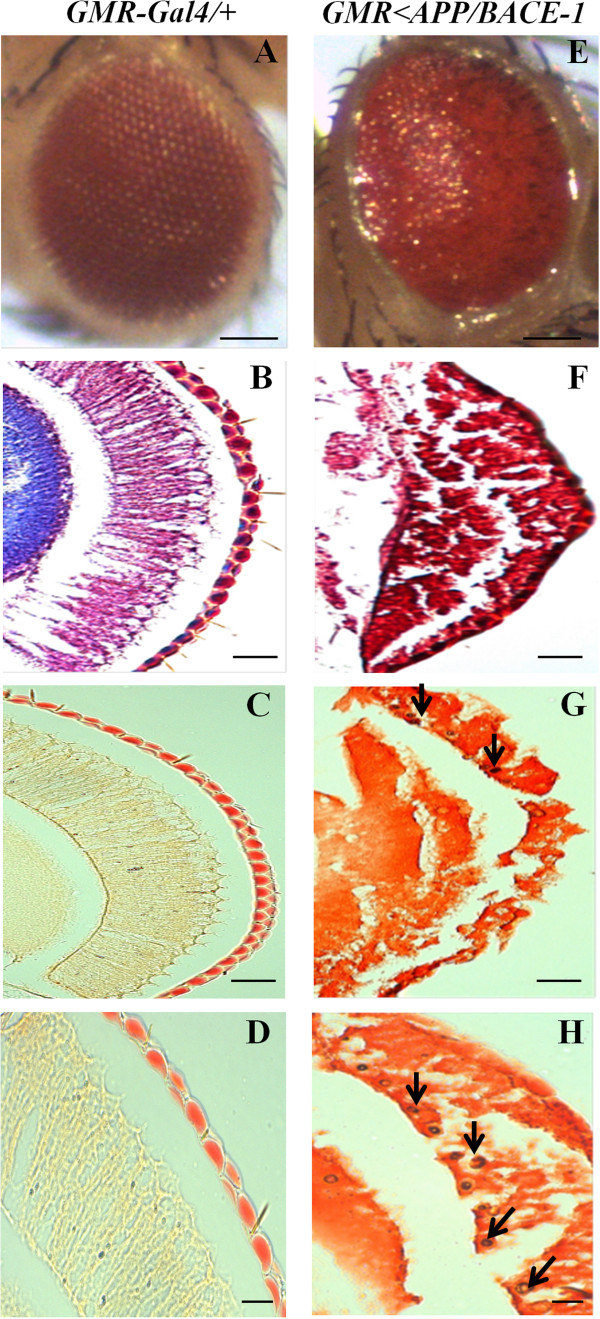
**Human APP and BACE-1 genes induced eye degeneration of transgenic fly under *GMR-Gal4 *driver strain.** Control *GMR-Gal4/+* flies showed normal and well-organized compounds eye **(A)**, this well-organized structure were stained by Cason’s trichome staining **(B)** and Congo red staining **(C** and **D)**. Transgenic flies *GMR < APP/BACE-1* presented rough and irregular compound eye **(E)**, damages of retina were detected by Cason’s trichome staining **(F)** and amyloid depositions were observed in flies’ compound eyes by Congo red staining marked by arrows **(G** and **H)**. Scale bar, 50 μm.

**Figure 4:**
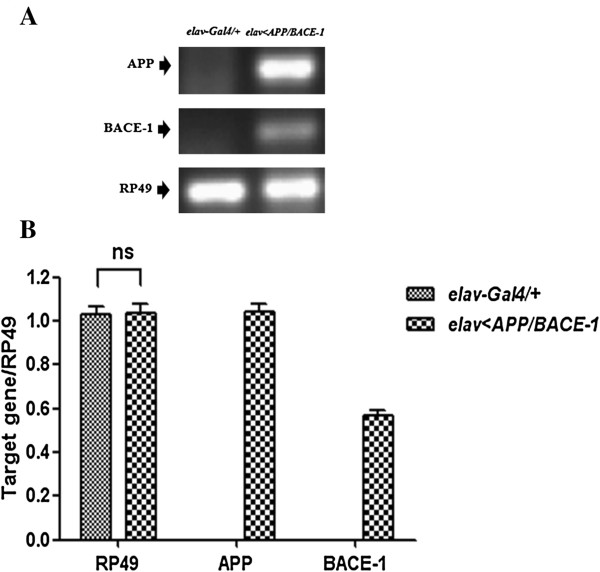
**mRNA expression level of transgenic flies, *elav-Gal4/+ *as control fly.** Target gene human APP and BACE-1 electrophoresis results **(A)** and semi-quantitative RT-PCR analysis of human APP and BACE-1 genes mRNA expression level **(B)**. Gene RP49 was used as the reference gene to normalize mRNA amount. For RP49 mRNA expression amount, there was no significant difference between control flies and transgenic flies, however, mRNA of target genes human APP and BACE-1 were not expressed in control flies. ns: no significant difference. Each bar represents standard error.

The GAL4/UAS system was employed for the overexpression of desired genes in a specific tissue of the fly. In our experiment, human APP and BACE-1 genes induced eye degeneration of transgenic fly under *GMR-Gal4* driver strain. Control *GMR-Gal4/+* flies showed normal and well-organized compounds eye (Figure 3A), well-organized structure and normal retina were stained by Cason’s trichome staining (Figure 3B) and Congo red staining (Figure 3C and D). However, human APP and BACE-1 co-expression flies presented rough and irregular compound eye ( Figure 3E), damages of retina were showed clearly by Cason’s trichome staining (Figure 3F), and amyloid depositions were observed by Congo red staining marked by with arrows (Figure 3G and H). APP and BACE-1 were expressed in the nervous system under *elav-GAL4* driver strain. We reconfirmed the expression of APP and BACE-1 genes in the transgenic flies with semi-quantitative RT-PCR analysis (Figure 4). Target genes electrophoresis results were observed (Figure 4A), Gene RP49 was used as the reference gene to normalize mRNA amount, human APP and BACE-1 genes showed high expression amount in transgenic fly, however, no expression in control fly (Figure 4B). In conclusion, trans-human APP and BACE-1 fly can be used as a reliable AD model.

### Effects of curcuminoids on the eye morphology

**Figure 5:**
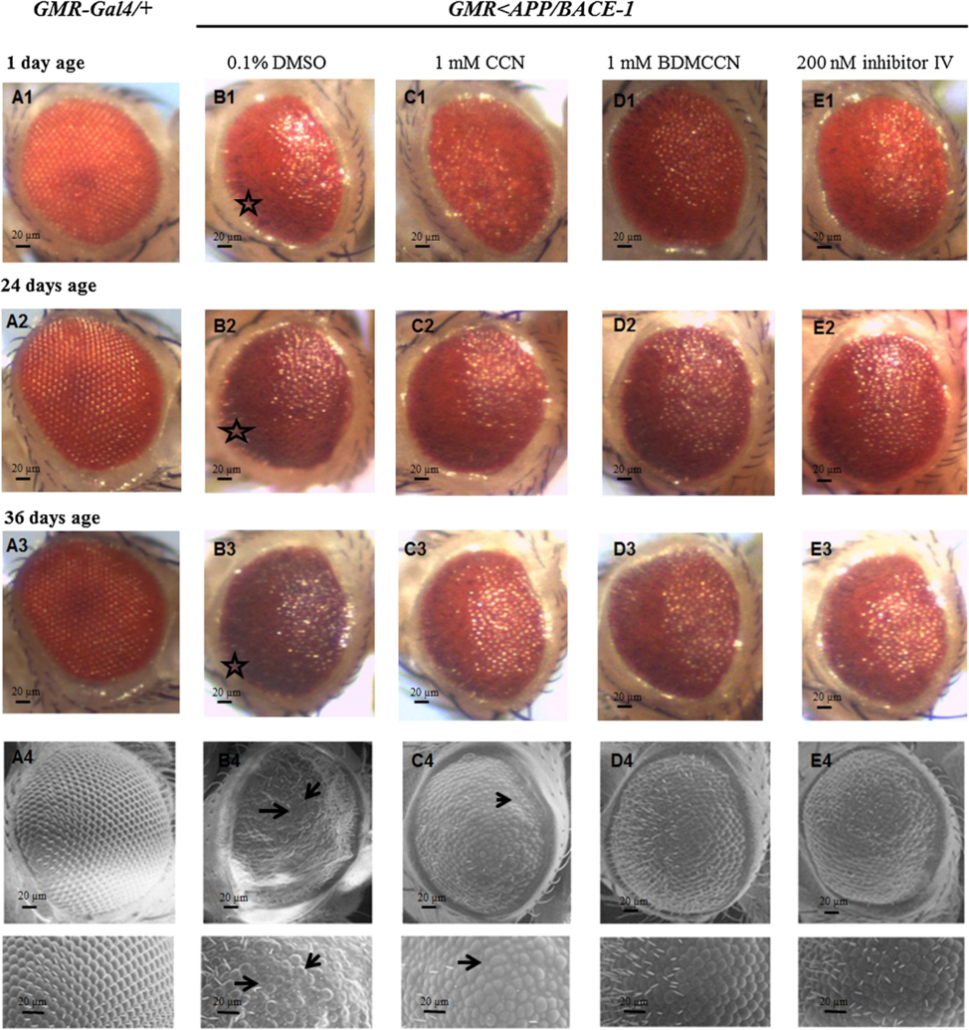
**Rough eye phenotype associated with *GMR < APP/BACE-1 Drosophila *eye development observed by light microscope and scanning electron microscope.** Light micrographs of *GMR < GAL4* flies **(A1-A3)**, *GMR < APP/BACE-1* cultured on 0.1% DMSO **(B1-B3)**, 1 mM curcumin **(C1-C3)**, 1 mM bisdemethoxycurcumin **(D1-D3)**, and 200 nM β-secretase Inhibitor IV **(E1-E3)**. Scanning electron micrographs of day 36 *GMR < GAL4* flies **(A4)** and *GMR < APP/BACE-1* cultured on different media **(B4-E4)**. Stars indicate ommatidia atrophia.

Morphological defects in flies expressing APP/BACE-1 in compound eye (*GMR > Gal4, UAS-APP, UAS-BACE-1 or GMR > APP/BACE-1*) were first examined. Control carrying *GMR-GAL4* alone showed wild-type eye morphology (Figure 5A). In contrast, *GMR > APP/BACE-1* developed ommatidia atrophia at the edge of compound eye (marked with star in Figure 5B). Next, we cultured *GMR > APP/BACE-1* flies in media supplemented with 1 mM CCN, 1 mM BDMCCN or 200 nM Inhibitor in 0.1% DMSO during entire developmental stages. Even on 1 day post-eclosion, the edge atrophia was already ameliorated in the flies cultured in CCN- (Figure 5C1) or BDMCCN-media (Figure 5D1), compared with the vehicle (0.1% DMSO) control (Figure 5B1). The manifestation of ommatidia atrophia was also considerably reduced in Inhibitor IV-fed flies (Figure 5E1). However, any of tested curcuminoids and Inhibitor IV failed to suppress completely the eye degeneration phenotype observed in *GMR > APP/BACE-1*. Nevertheless, protective effects of CCN, BDMCCN and Inhibitor IV remained evident in 24 day- and 36-day old flies (Figure 5).

To compare the protective activities of curcuminoids in high resolution, we examined compound eyes of *GMR > APP/BACE-1* treated with each compound using scanning electron microscope (SEM). Control flies (*GMR-GAL4*/+) showed smooth appearance of the eye without any defects of ommatidia size and bristles (Figure 5A4). In contrast, *GMR > APP/BACE-1* flies showed varying degrees of eye disorganization. The eye of flies treated with the vehicle showed the strongest phenotypes characterized by absence of ommatidial bristles and fusion of ommatidia (marked with arrows) (Figure 5B4). The rough eye phenotype was suppressed partially in 1 mM CCN-fed flies, but most ommatidial bristles were still absent and the size of some ommatidia reduced (arrows in Figure 5C4). Remarkably, protective potency of 1 mM BDMCCN (Figure 5D4) was comparable to that of 200 nM inhibitor IV (Figure 5E4).

### Effects of curcuminoids on climbing behaviors

**Figure 6:**
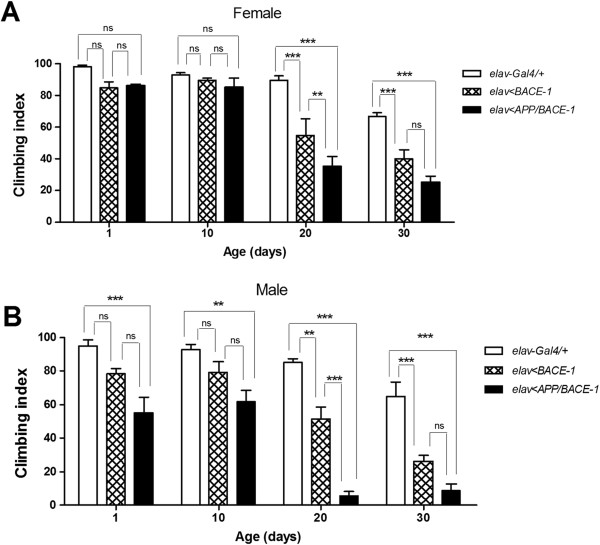
**Climbing behavior of females (A) and males (B) from *elav-Gal4/+, elav < BACE-1, *and *elav < APP/BACE-1 *fed standard media.** (*n* = 50–100 flies ***: *p* < 0.001, **: *p* < 0.01, ns: no significant difference). Each bar represents standard error.

Most of neurodegenerative diseases including Alzheimer’s disease are characterized by age-dependent deterioration in locomotory coordination. In *Drosophila* model, the locomotory coordination can be quantified by the negative geotaxis assay, which takes advantage of fly’s innate tendency to climb against gravity after gentle tapping. In the assay, control flies cultured on standard media (*elav-Gal4/+*) showed a clear age-dependent reduction in the climbing indices, for example measured in females as 98%, 93%, 89% and 66% in 1, 10, 20 and 30 days after eclosion, respectively (Figure 6A). Further, expression of BACE-1 with or without its substrate APP in the nervous system resulted in even stronger age-dependent locomotory deterioration in both genders (Figure 6). The climbing defect was slightly more pronounced in males than in females, there was virtually no climbing activity scored in *elav > APP/BACE-1* males from 20 days after eclosion (Figure 6B).

**Figure 7:**
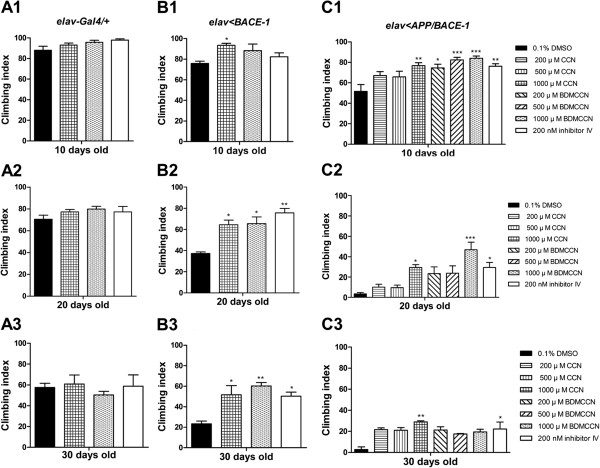
**Effect of curcumin, bisdemethoxycurcumin, and β-secretase Inhibitor IV supplementation on climbing behavior of 10, 20, and 30 days old male flies from *elav-Gal4/+ *(A), *elav < BACE-1 *(B), and *elav < APP/BACE-1 *(C) (*n*** **= 50–100 flies.** (***: *p* < 0.001, **: *p* < 0.01, *: *p* < 0.05). Each bar represents standard error.

Then, we asked whether two curcuminoids and Inhibitor IV BACE-1 blockers could rescue climbing defects in males expressing BACE-1 alone (*elav > BACE-1*) of three different age groups (10, 20 and 30 days post-eclosion). In 10 days after eclosion, all tested compounds except 1 mM CCN did not significantly improve climbing ability, probably because overall behavioral defect was not pronounced in this age group. In contrast, tested 20 and 30 days after eclosion, *elav > BACE-1* males cultured in media containing 1 mM CCN, 1 mM BDMCCN, or 200 nM Inhibitor IV showed significantly improved climbing indices, compared with those cultured in vehicle control (0.1% DMSO) media (Figure 7B1-B3).

Subsequently, we carried out analogous experiments with *elav > APP/BACE-1* males, which show much severe age-dependent progression of locomotory defects. In 10 days post-eclosion, males cultured with CCN (1 mM only), BDMCCN (200 μM, 500 μM, and 1 mM), or inhibitor IV (200 nM) showed less severe impairments in the climbing ability compared with flies cultured in vehicle control (Figure 7C1). In 20 days post-eclosion, virtually no *elav > APP/BACE-1* males can climb against gravity, due to poor movement coordination. However, dietary supplement of 1 mM CCN, 1 mM BDMCCN, or 200 nM Inhibitor IV delayed age-dependent progression of locomotory defects, and improved climbing ability compared with vehicle control (Figure 7C2). The climbing ability was also partially rescued with 1 mM CCN- and 200 nM Inhibitor IV in 30 days post-eclosion males (Figure 7C3).

### Effects of curcuminoids on life span and feeding

**Figure 8:**
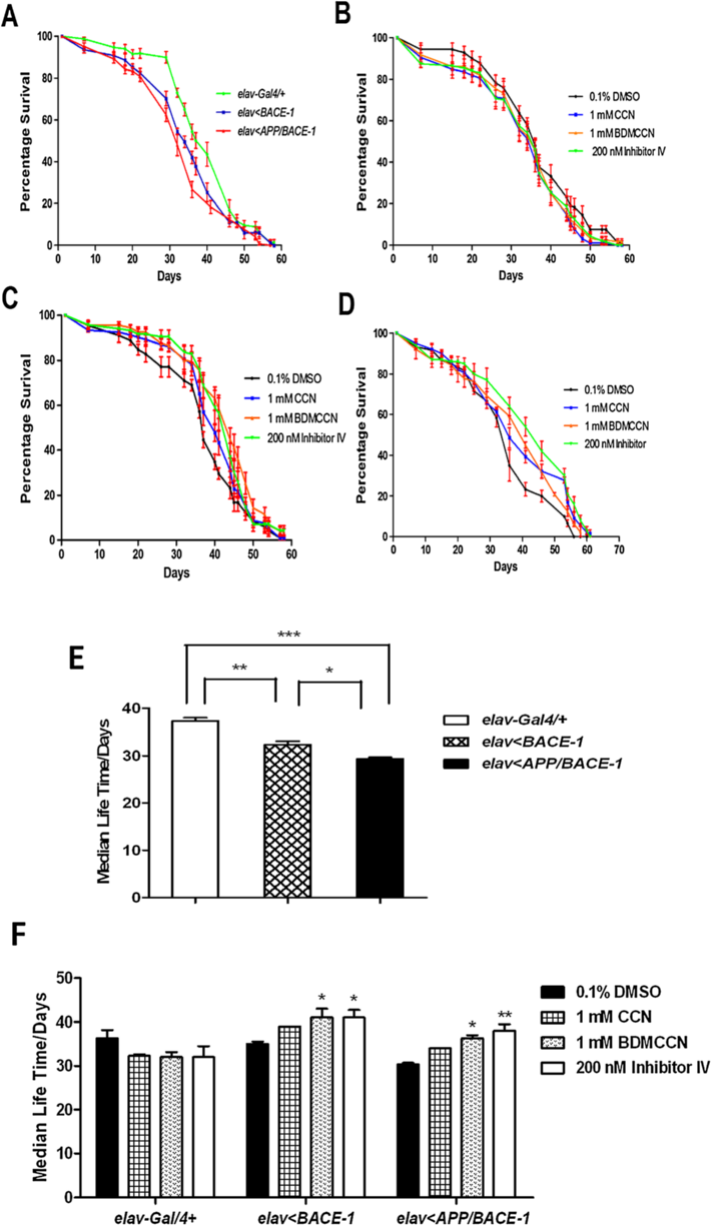
**Effect of curcumin, bisdemethoxycurcumin, and β-secretase Inhibitor IV supplementation on longevity of *elav-Gal4/+, elav < BACE-1, *and *elav < APP/BACE-1 *flies.** There was no significant difference in the longevity among three different genotype flies **(A)**. Compound supplementation did not affect longevity of flies *elav-Gal4/+***(B)**, *elav < BACE-1***(C)**, and *elav < APP/BACE-1***(D)**. Median life time of three genotype flies cultured on standard medium (n = 200 flies per group. ***: *p* < 0.001, **: *p* < 0.01, *: *p* < 0.05) **(E)**. Median life time of three genotype flies fed on curcuminoids and BACE-1 Inhibitor IV supplementation media (n = 200 flies per group. **: *p* < 0.01, *: *p* < 0.05) **(F)**. Each bar represents standard error.

Previously, it was reported that expression of BACE-1 with APP reduced life span of adult flies [10]. Thus, we asked whether prolonged exposure of *elav > BACE-1* or *elav > APP/BACE-1* to curcuminoids increases life span. The life span of the male flies fed on standard media did not differ significantly from each other (Figure 8A). Supplementation of 1 mM CCN, 1 mM BDMCCN, and 200 nM inhibitor IV did not affect longevity of *elav-Gal4/+* flies (Figure 8B), *elav < BACE-1* flies (Figure 8C), and *elav < APP/BACE-1* flies (Figure 8D). Interestingly, cultured in vehicle control, *elav > BACE-1* males showed significantly shorter median life time (T_1/2_, 33 days) than control (T_1/2_, 37 days in *elav-Gal4/+*). Expression of APP together with BACE-1 (*elav > APP/BACE-1*) reduced T_1/2_ even further to 30 days (Figure 8E). Culturing flies in curcuminoids or Inhibitor IV increased T_1/2_ in *elav > BACE-1* and *elav > APP/BACE-1*, but not in control lacking *UAS-BACE-1* (*elav-Gal4/+*). 1 mM BDMCCN increased T_1/2_ as much as 200 nM Inhibitor IV did in *elav > BACE-1* flies (42 vs. 41 days). 1 mM BDMCCN also significantly rescue median life time in *elav > APP/BACE-1* flies (T_1/2_, 36 days), but 1 mM CCN did not (Figure 8F).

**Figure 9:**
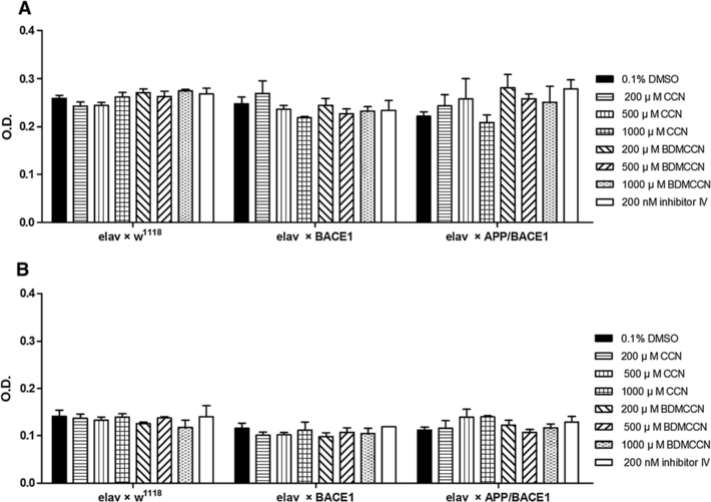
**Effect of curcumin, bisdemethoxycurcumin, and β-secretase Inhibitor IV supplementation on adult flies feeding behavior of *elav-Gal4/+, elav < BACE-1, *and *elav < APP/BACE-1 *female (A) and male (B) flies.***n* = 45 flies. Each bar represents standard error.

Because feeding behavior is one of the essential factors determining longevity of flies and other animals [35], we examined the effects of compounds on amount of food intake in adults expressing APP/BACE-1 in the nervous system (*elav > APP/BACE-1*). Irrespective of compounds and concentrations examined, CCN, BDMCCN, and Inhibitor IV did not affect amount of feeding in any of tested genotypes (Figure 9). This finding indicates that protective effects of BDMCCN and Inhibitor IV on longevity are not attributed to their possible anorectic effects.

## Discussion

Plants and their constituents are a potential for AD therapy because some are selective and biodegrade to nontoxic products. Various compounds such as alkaloids, phenolics, and terpenoids, exist in plants and jointly or independently they contribute to BACE-1 inhibition [6,7]. Human BACE-1 inhibitory activity has been reported for catechins (EGCG, (−)-epicatechin gallate) [22]; chromone glycosides (e.g. aloeresin D) [29]; isoflavones (bavachinin, neocorylin) and chalcone flavonoids (e.g. bavachromene, bavachalcone) [36]; (+)-vitisinol E, (+)-ampelopsin A, and (+)-vitisin [37]; furanocoumarins (imperatorin, (+)-byakangelicol) [38]; amentoflavone-type biflavonoids (e.g. 2,3-dihydroamentoflavone, 2,3-dihydro-6-methylginkgetin) [39]; resveratrol and its derivatives [40]. In the current study, the active principles of *C. longa* rhizome were determined to be the diarylalkyls CCN (**1**), DMCCN (**2**), and BDMCCN (**3**). BDMCCN was the most potent BACE-1 inhibitory constituent, followed by DMCCN and CCN. Nevertheless, all of the individual compounds were less inhibitory than Inhibitor IV.

QSAR of BACE-1 inhibitors have been well reviewed previously [11,41]. BACE-1 inhibitory activity of 10 catechins and reported that the inhibitory activity seemed to be related to the pyrogallol moiety on C-2 and/or C-3 catechin skeleton, whereas the stereochemistry of C-2 and C-3 did not have the inhibitory activity [42]. In the current study, absence of methoxy groups in the phenyl rings of CCN increased the BACE-1 inhibitory activity. In particular, absence of two methoxy groups (BDMCCN) significantly increased BACE-1 inhibitory activity. However, THCCN was less potent at inhibiting BACE-1 inhibitory activity than CCN, indicating that the double bonds appear to be essential for the enzyme inhibitory activity. This current finding indicates that structural characteristics, such as degrees of saturation, carbon skeleton, types of functional group, and hydrophobicity rather than MW appear to play a role in determining the BACE-1 inhibitory activity. Previous study [42] also reported that BDMCCN exhibited the most potent inhibitory action on BACE-1 mRNA level, followed by DMCCN and CCN.

Although multiple pathogenetic factors such as Aβ and tau aggregation, excessive metal ions, oxidative stress, acetylcholine level, and increased BACE-1 activity have been suggested for AD, lifestyles and genetic factors also are associated with AD development [43]. The potency of feeding curcumin as a drug candidate to alleviate Aβ toxicity in transgenic *Drosophila* was also studied [18]. They reported that the longevity and the locomotor activity of five different AD model genotypes showed up to 75% improved lifespan and activity for curcumin-fed flies and any decrease in the amount of Aβ deposition following curcumin treatment was not observed. In the current study, we showed prolonged exposure to either CCN or BDMCCN could rescue morphological defects observed in flies expressing APP and BACE-1 in compound eyes (*GMR > APP/BACE-1*). In addition, dietary supplement of CCN or BDMCCN also improved movement coordination significantly in *elav > BACE-1* and *elav > APP/BACE-1* flies, but not in control. Lastly, both BDMCCN and Inhibitor IV increased median life time of *elav > BACE-1* and *elav > APP/BACE-1* flies. This finding along with BACE-1 inhibitory action indicates that materials derived from *C. longa* rhizome root may hold promise for the development of novel and effective anti-AD products.

## Conclusions

*C. longa* rhizome-derived preparations containing curcuminoids described could be useful as sources of potential therapeutics or lead molecules for prevention or treatment of AD. For practical use of *C. longa* rhizome-derived materials as novel anti-AD products to proceed, further research is needed to establish their human safety and whether this activity could be exerted *in vivo* after consumption of the product by humans. Historically, the rhizome has been commonly used as a spice in curries and other South Asian and Middle Eastern cuisine, flavoring agents, and coloring agents [44]. In addition, their anti-AD modes of action need to be established and formulations for improving anti-AD potency and stability need to be developed because of the poor bioavailability and stability in solution [18].

## Abbreviations

AD: Alzheimer’s disease; BACE-1: β-amyloid precursor cleavage enzyme; CCN: Curcumin; DMCCN: Demethoxycurcumin; BDMCCN: Bisdemethoxycurcumin; Aβ: Amyloid β; APP: Amyloid precursor protein; AChE: Acetylcholinesterase; EGCG: (−)-epigallocatechin gallate; FRET: Fluorescence resonance energy transfer; QSAR: Quantitative structure–activity relationship; T_1/2_: median survival time; DMSO: Dimethyl sulfoxide.

## Competing interest

The authors declare that they have no competing interests.

## Authors’ contributions

XW and YJA conceived and designed the experiments, interpreted the data and drafted the manuscript. XW participated in the experiment. SBL, MYJ, JRK, YJK, and HWK offered valuable suggestion and helped in drafting of manuscript. All authors read and approved the final manuscript.

## Pre-publication history

The pre-publication history for this paper can be accessed here:

http://www.biomedcentral.com/1472-6882/14/88/prepub

## Supplementary Material

Additional file 1: Figure S1 EI-Mass spectrum of compound 1. Click here for file

Additional file 2: Figure S2 1H-NMR spectrum of compound 1. Click here for file

Additional file 3: Figure S3 13C-NMR spectrum of compound 1. Click here for file

Additional file 4: Figure S4 EI-Mass spectrum of compound 2. Click here for file

Additional file 5: Figure S5 1H-NMR spectrum of compound 2. Click here for file

Additional file 6: Figure S6 13C-NMR spectrum of compound 2. Click here for file

Additional file 7: Figure S7 EI-Mass spectrum of compound 3. Click here for file

Additional file 8: Figure S8 1H-NMR spectrum of compound 3. Click here for file

Additional file 9: Figure S9 13C-NMR spectrum of compound 3. Click here for file

## References

[B1] Ferri C Prince M Brayne C Brodaty H Flatiglioni L Ganguli M Hall K Hasegawa K Hendrie H Huang Y Jorm A Mathers C Menezes P Rimmer E Scazufca M Global prevalence of dementia: a Delphi consensus study Lancet 2005 366 2112 2117 10.1016/S0140-6736(05)67889-0 16360788 PMC2850264

[B2] Kalaria R Maestre GE Arizaga R Friedland RP Galasko D Hall K Luchsinger JA Ogunniyi A Perry EK Potocnik F Prince M Stewart R Wimo A Zhang ZX Antuono P Alzheimer’s disease and vascular dementia in developing countries: prevalence, management, and risk factors Lancet Neurol 2008 7 812 826 10.1016/S1474-4422(08)70169-8 18667359 PMC2860610

[B3] Brookmeyer R Johnson E Ziegler-Graham K Arrighi HM Forecasting the global burden of Alzheimer’s disease Alzheimers Dement 2007 3 186 191 10.1016/j.jalz.2007.04.381 19595937

[B4] Hardy J Selkoe DJ The amyloid hypothesis of Alzheimer’s disease: progress and problems on the road to therapeutics Science 2002 297 353 356 10.1126/science.1072994 12130773

[B5] Suh YH Checler F Amyloid precursor protein, presenilins, and alpha-synuclein: molecular pathogenesis and pharmacological applications in Alzheimer’s disease Pharmacol Rev 2002 54 469 525 10.1124/pr.54.3.469 12223532

[B6] Ghosh AK Gemma S Tang J beta-Secretase as a therapeutic target for Alzheimer’s disease Neurotherapeutics 2008 5 399 408 10.1016/j.nurt.2008.05.007 18625451 PMC2963069

[B7] Mancini F De Simone A Andrisano V Bata-secrtase as a target for Alzheimer’s disease drug discovery: an overview of in vitro methods for characterization of inhibitors Anal Bioanal Chem 2011 400 1979 1996 10.1007/s00216-011-4963-x 21503735

[B8] Greeve I Kretzschmar D Tschäpe JA Beyn A Brellinger C Schweizer M Nitsch RM Reifegerste R Age-dependent neurodegeneration and Alzheimer amyloid plaque formation in transgenic Drosophila J Neurosci 2004 24 3899 3906 10.1523/JNEUROSCI.0283-04.2004 15102905 PMC6729409

[B9] Crowther DC Kinghorn KJ Miranda E Page R Curry JA Duthie FA Gubb DC Lomas DA Intraneuronal Abeta, non-amyloid aggregates and neurodegeneration in a Drosophila model of Alzheimer’s disease Neuroscience 2005 132 123 135 10.1016/j.neuroscience.2004.12.025 15780472

[B10] Chakraborty R Vepuri V Mhatre SD Paddock BE Miller S Michelson SJ Delvadia R Desai A Vinokur M Melicharek DJ Utreja S Khandelwal P Ansaloni S Goldstein LE Moir RD Lee JC Tabb LP Saunders AJ Marenda DR Characterization of a Drosophila Alzheimer’s disease model: pharmacological rescue of cognitive defects PLoS One 2011 6 6 e20799 10.1371/journal.pone.0020799 21673973 PMC3108982

[B11] Thompson LA Bronson JJ Zusi FC Progress in the discovery of BACE inhibitors Curr Pharm Des 2005 11 3383 3404 10.2174/138161205774370825 16250843

[B12] John V Human beta-secretase (BACE) and BACE inhibitors: progress report Curr Top Med Chem 2006 6 569 578 10.2174/156802606776743084 16712492

[B13] Erdogan Orhan I Current concepts on selected plant secondary metabolites with promising inhibitory effects against enzymes linked to Alzheimer’s disease Curr Med Chem 2012 19 2252 2261 22414107 10.2174/092986712800229032

[B14] Rates SMK Plants as source of drugs Toxicon 2001 39 603 613 10.1016/S0041-0101(00)00154-9 11072038

[B15] Raskin I Ribnicky DM Komarnytsky S Ilic N Poulev A Borisjuk N Brinker A Moreno DA Ripoll C Yakoby N O’Neal JM Cornwell T Pastor I Fridlender B Plants and human health in the twenty-first century Trends Biotechnol 2002 20 522 531 10.1016/S0167-7799(02)02080-2 12443874

[B16] Mukherjee PK Kumar V Mal M Houghton PJ Acetylcholinesterase inhibitors from plants Phytomedicine 2007 14 289 300 10.1016/j.phymed.2007.02.002 17346955

[B17] Lee KS Lee BS Semnari S Avanesian A Um CY Jeon HJ Seong KM Yu K Min KJ Jafari M Curcumin extends life span, improves health span, and modulates the expression of age-associated aging genes in *Drosophila melanogaster* Rejuvenation Res 2010 13 561 570 10.1089/rej.2010.1031 20645870

[B18] Caesar I Jonson M Nilsson KPR Thor S Hammarström P Curcumin promotes A-beta fibrillation and reduces neurotoxicity in transgenic *Drosophila* PLoS One 2012 7 2 e31424 10.1371/journal.pone.0031424 22348084 PMC3278449

[B19] Tang E Eisenbrand G Chinese Drugs of Plant Origin 1992 New York: Springer

[B20] Araújo CC Leon LL Biological activities of *Curcuma longa* L Mem Inst Oswaldo Cruz 2001 96 723 728 10.1590/S0074-02762001000500026 11500779

[B21] Kuhn MA Winston D Herbal Therapy and Supplements: A Scientific & Traditional Approach 2001 New York: Lippincott 330 335

[B22] Jeon SY Bae K Seong YH Song KS Green tea catechins as a BACE1 (β-secretase) inhibitor Bioorg Med Chem Lett 2003 13 3905 3908 10.1016/j.bmcl.2003.09.018 14592472

[B23] Stachel SJ Coburn CA Steele TG Jones KG Loutzenhiser EF Gregro AR Structure-based design of potent and selective cell-permeable inhibitors of human β-secretase (BACE-1) J Med Chem 2004 47 6447 6450 10.1021/jm049379g 15588077

[B24] Lv L Yang QY Zhao Y Yao CS Sun Y Yang EJ Song KS Mook-Jung I Fang WS BACE1 (beta-secretase) inhibitory chromone glycosides from *Aloe vera* and *Aloe nobilis* Planta Med 2008 74 540 545 10.1055/s-2008-1074496 18543151

[B25] Perumalsamy H Kim JR Oh SM Jung JW Ahn YJ Kwon HW Novel histopathological and molecular effects of natural compound pellitorine on larval midgut epithelium and anal gills of *Aedes aegypti* PLoS One 2013 8 11 e80226 10.1371/journal.pone.0080226 24260359 PMC3832413

[B26] Pirooznia SK Sarthi J Johnson AA Toth MS Chiu K Koduri S Elefant F Tip60 HAT Activity Mediates APP Induced Lethality and Apoptotic Cell Death in the CNS of a *Drosophila* Alzheimer’s Disease Model PLoS One 2012 7 7 e41776 10.1371/journal.pone.0041776 22848598 PMC3406101

[B27] Kwak YD Wang R Li JJ Zhang YW Xu H Liao FF Differential regulation of BACE1 expression by oxidative and nitrosative signals Mol Neurodegener 2011 6 17 10.1186/1750-1326-6-17 21371311 PMC3059281

[B28] Park YT Jeong J-y Lee M-j Kim K-i Kim T-H Kwon Y-d Lee C Kim Ok J An H-J MicroRNAs overexpressed in ovarian ALDH1-positive cells are associated with chemoresistance J Ovarian Res 2013 6 18 http://www.ovarianresearch.com/content/6/1/18 10.1186/1757-2215-6-18 23522567 PMC3637599

[B29] Hartman H Hayes TL Scanning electron microscopy of Drosophila J Hered 1971 62 41 44 5094716 10.1093/oxfordjournals.jhered.a108118

[B30] Park JH Jung JW Ahn YJ Kwon HW Neuroprotective properties of phytochemicals against paraquat-induced oxidative stress and neurotoxicity in *Drosophila melanogaster* Pesticide Biochem Physiol 2012 104 118 125 10.1016/j.pestbp.2012.07.006

[B31] Mahoney MB Singh CM Diggins LT Keefe D Lund E O’Neil P Sigel E Symonds J Villaluz A Ahlijanian MK Palfreyman MG Compound screening in a *Drosophila melanogaster* Alzheimer’s disease model using a behavioral readout Alzheimers Dement 2009 5 e11 e12

[B32] Bahadorani S Bahadorani P Phillips JP Hilliker AJ The effects of vitamin supplementation on *Drosophila* life span under normoxia and under oxidative stress J Gerontol A Biol Sci Med Sci 2008 63 35 42 10.1093/gerona/63.1.35 18245758

[B33] Min KJ Tatar M *Drosophila* diet restriction in practice: do flies consume fewer nutrients? Mech Ageing Dev 2006 2006 127 93 96 16256171 10.1016/j.mad.2005.09.004

[B34] Jayaprakasha GK Rao LJM Sakariah KK Improved HPLC method for the determination of curcumin, demethoxycurcumin, and bisdemethoxycurcumin J Agric Food Chem 2002 50 3668 3672 10.1021/jf025506a 12059141

[B35] Carvalho Gil B Kapahi P Benzer S Compensatory ingestion upon dietary restriction in *Drosophila melanogaster* Nat Methods 2005 2 813 815 10.1038/nmeth798 16278649 PMC2745347

[B36] Choi YH Yon GH Hong KS Yoo DS Choi CW Park WK Kong JY Kim YS Ryu SY *In vitro* BACE-1 inhibitory phenolic components from the seeds of *Psoralea corylifolia* Planta Med 2008 74 1405 1408 10.1055/s-2008-1081301 18666047

[B37] Choi YH Yoo MY Choi CW Cha MR Yon GH Kwon DY Kim YS Park WK Ryu SY A new specific BACE-1 inhibitor from the stembark extract of Vitis vinifera Planta Med 2009 75 537 540 10.1055/s-0029-1185311 19184970

[B38] Marumoto S Miyazawa M beta-secretase inhibitory effects of furanocoumarins from the root of *Angelica dahurica* Phytother Res 2010 24 510 513 20041416 10.1002/ptr.2967

[B39] Sasaki H Miki K Kinoshita K Koyama K Juliawaty LD Achmad SA Hakim EH Kaneda M Takahashi K β-Secretase (BACE-1) inhibitory effect of biflavonoids Bioorg Med Chem Lett 2010 20 4558 4560 10.1016/j.bmcl.2010.06.021 20598535

[B40] Choi CW Choi YH Cha MR Kim YS Yon GH Hong KS Park WK Kim YH Ryu SY In vitro BACE1 inhibitory activity of resveratrol oligomers from the seed extract of *Paeonia lactiflora* Planta Med 2011 77 374 637 10.1055/s-0030-1250370 20890809

[B41] John V Beck JP Bienkowski MJ Sinha S Heinrikson RL Human β-secretase (BACE) and BACE inhibitors J Med Chem 2003 46 4625 4630 10.1021/jm030247h 14561080

[B42] Liu H Li Z Qiu D Gu Q Lei Q Mao L The inhibitory effects of different curcuminoids on β-amyloid protein, β-amyloid precursor protein and β-site amyloid precursor protein cleaving enzyme 1 in swap HEK293 cells Neurosci Lett 2010 485 83 88 10.1016/j.neulet.2010.08.035 20727383

[B43] Ji H Zhang H Multipotent natural agents to combat Alzheimer’s disease. Functional spectrum and structural features Acta Pharmacol Sin 2008 29 143 151 10.1111/j.1745-7254.2008.00752.x 18215342

[B44] Itokawa H Shi Q Akiyama T Morris-Natschke SL Lee KH Recent advances in the investigation of curcuminoids Chin Med 2008 3 11 doi: 10.1186/1749-8546-3-11 10.1186/1749-8546-3-11 18798984 PMC2576304

