# Supplementary figures and images for: Effects of curcuminoids identified in rhizomes of Curcuma longa on BACE-1 inhibitory and behavioral activity and lifespan of Alzheimer’s disease Drosophila models
Source: BMC Complement Altern Med. 2014 Mar 5;14:88. doi: 10.1186/1472-6882-14-88 (PMC3946151; doi:10.1186/1472-6882-14-88)

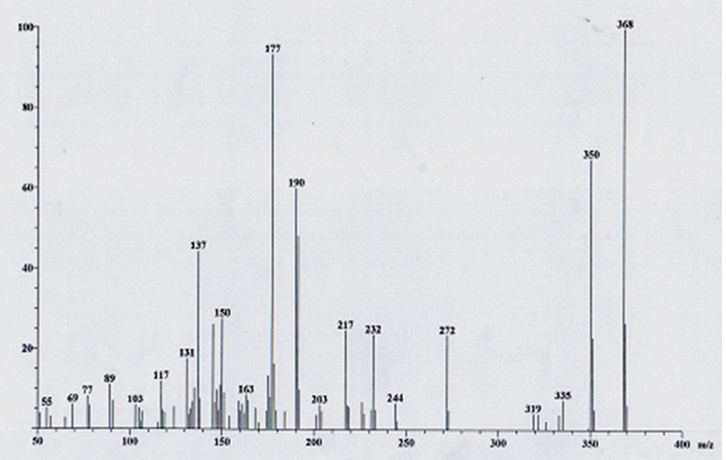

Supplement: Additional file 1: Figure S1 — EI-Mass spectrum of compound 1. [file 1472-6882-14-88-S1.jpg]

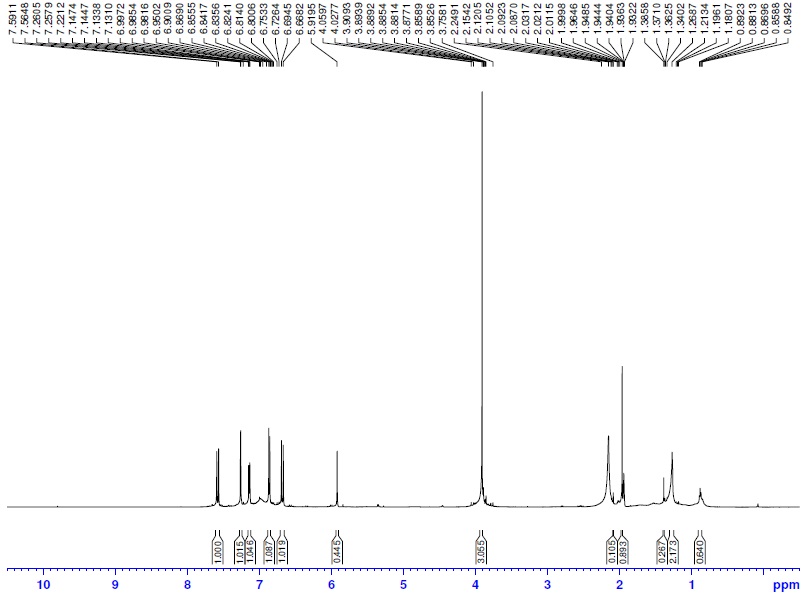

Supplement: Additional file 2: Figure S2 — 1H-NMR spectrum of compound 1. [file 1472-6882-14-88-S2.jpg]

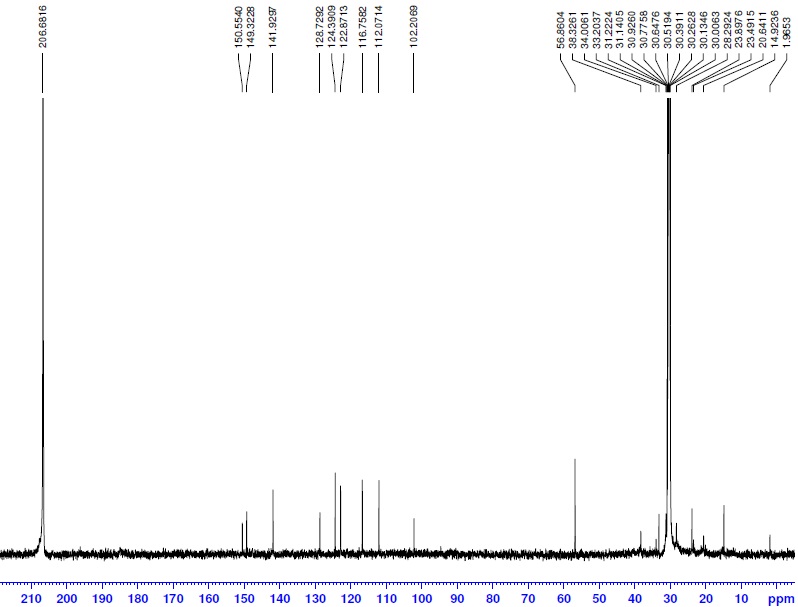

Supplement: Additional file 3: Figure S3 — 13C-NMR spectrum of compound 1. [file 1472-6882-14-88-S3.jpg]

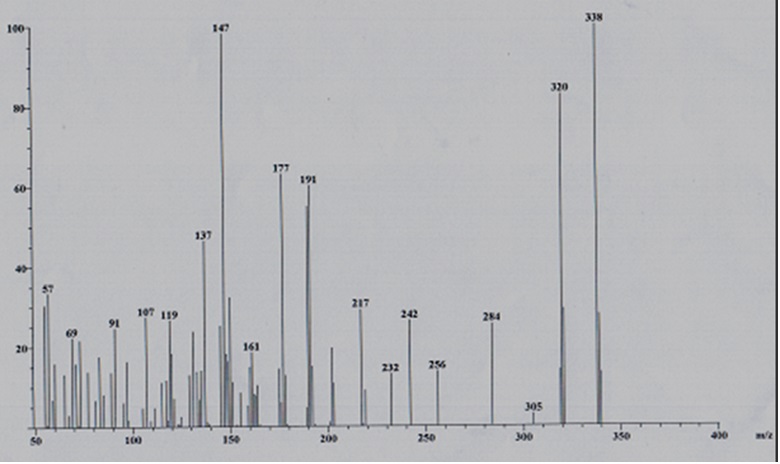

Supplement: Additional file 4: Figure S4 — EI-Mass spectrum of compound 2. [file 1472-6882-14-88-S4.jpg]

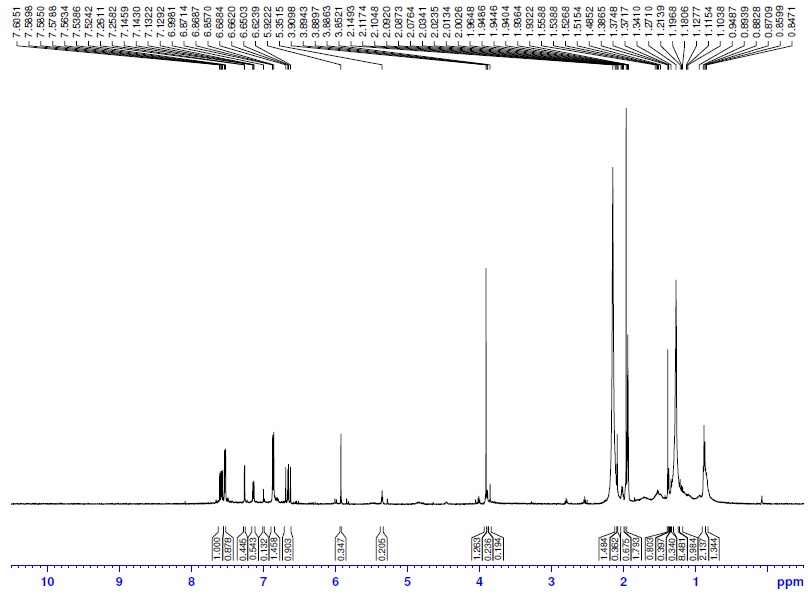

Supplement: Additional file 5: Figure S5 — 1H-NMR spectrum of compound 2. [file 1472-6882-14-88-S5.jpg]

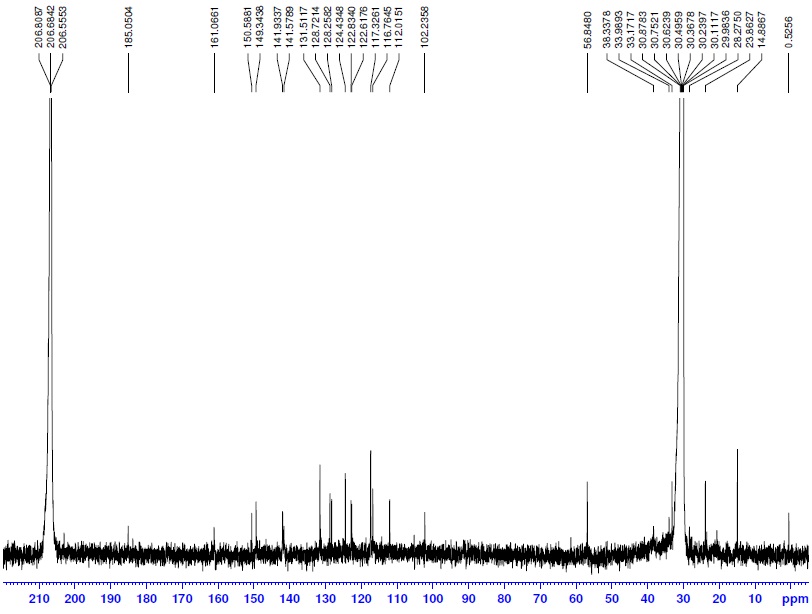

Supplement: Additional file 6: Figure S6 — 13C-NMR spectrum of compound 2. [file 1472-6882-14-88-S6.jpg]

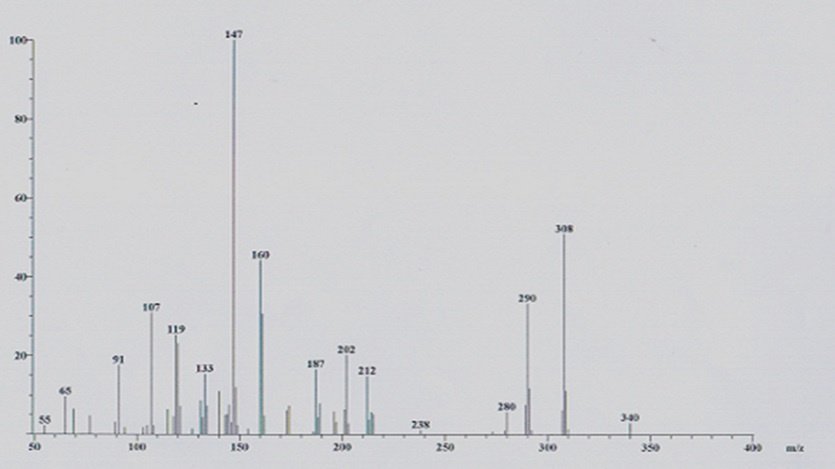

Supplement: Additional file 7: Figure S7 — EI-Mass spectrum of compound 3. [file 1472-6882-14-88-S7.jpg]

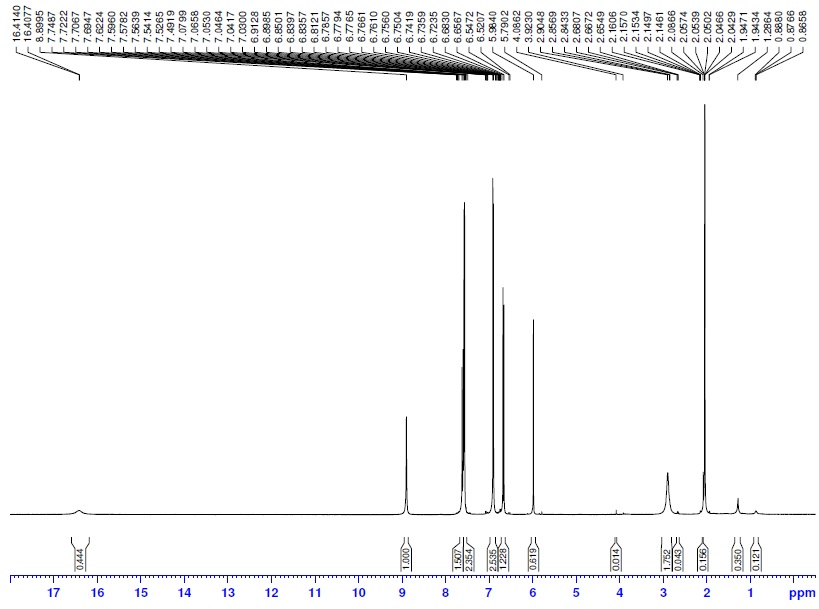

Supplement: Additional file 8: Figure S8 — 1H-NMR spectrum of compound 3. [file 1472-6882-14-88-S8.jpg]

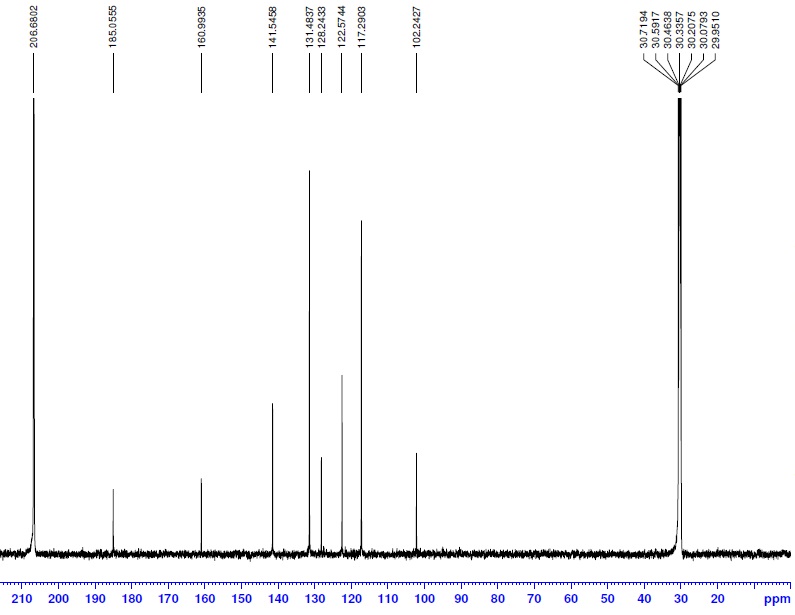

Supplement: Additional file 9: Figure S9 — 13C-NMR spectrum of compound 3. [file 1472-6882-14-88-S9.jpg]
